# Impact of GO Chemical Composition on the Performance of Humidity Sensors

**DOI:** 10.1021/acsomega.5c04175

**Published:** 2025-07-18

**Authors:** Nayton C. Vicentini, Alessandro H. Lima, Giovanni R. Carvalho, Camila T. Tavares, Anne C. P. Fernandes, Clemilda C. S. Cunha, Joyce R. Araújo, Sanair M. S. Palheta, Benjamin Fragneaud, Indhira O. Maciel, Cristiano Legnani, Welber G. Quirino

**Affiliations:** † Nanoscience and Nanotechnology Group − Nano, Physics Department, 28113 UFJF, Juiz de Fora-MG 36036-900, Brazil; ‡ Materials Metrology Division, National Institute of Metrology, Quality and Technology − INMETRO, Duque de Caxias-RJ 25250-020, Brazil

## Abstract

Graphene oxide (GO), a structurally defective 2D carbon nanomaterial, is very promising for relative humidity (RH) sensing applications due to the presence of diverse oxygenated functional groups (OFGs) in its structure. The characteristics of GO, such as flake size, degree of oxidation and exfoliation, permanent structural defects, and chemical composition, directly impact the RH detection performance of GO. In this work, we investigated the performance of resistive RH sensors based on three types of GO, prepared using modifications of the Hummers’ method, namely, GO-I, GO-II, and GO-III, having different chemical composition, degree of oxidation, as well as different levels of permanent structural defects (carbon vacancies) at the basal plane. GO-based RH sensors were fabricated by drop-casting GO suspensions onto aluminum interdigitated electrodes thermally evaporated onto glass substrates. Among the three characterized RH sensors, GO-II-based devices showed superior performance, with a sensitivity of 2113 ± 2% compared to 1592 ± 1% for GO-I and 388.1 ± 0.1% for GO-III, respectively. All GO sensors demonstrated rapid response and recovery times (ca. 2 and 3 s). Our results indicate that improved quantities of highly polar OFGs, such as carbonyl and hydroxyl groups, and the associated permanent structural defects in GO-II, significantly improved its RH sensing properties. In addition, all GO-based RH sensors can be operated at only 0.1 V, making them suitable for integration into low-power systems.

## Graphical abstract

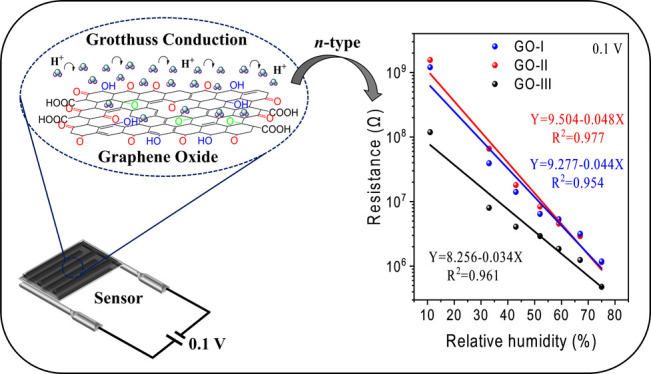

## 1 Introduction

Relative humidity (RH) sensing is of great importance in various fields, including food and beverage quality monitoring, environmental, comfort and health, meteorology, industry, medicine and pharmaceutical manufacturing, among others.^1–7^ Hence, the development of sensors for accurate and reliable measurement of RH is desirable and important. For commercial applications, such devices must be easy to manufacture on a large scale and at an affordable cost, as well as be easy to maintain and have long-term stability. Additionally, it is imperative that the sensing materials exhibit high sensitivity to even small changes in RH and that the sensor displays a fast response and recovery time over a wide humidity range.

Recently, carbon nanomaterials (CNMs) have been extensively studied due to their excellent physicochemical properties.^3,8–11^ Among CNMs, GO has received considerable attention, and it is now a commercial material employed in numerous applications. GO contains various OFGs in its structure, such as epoxide (−C–O–C−), hydroxyl (−OH), carbonyl (−C=O), and carboxylic acid groups (−COOH), covalently bonded to its basal plane and edges.^12–14^ These OFGs provide GO with high hydrophilicity,^15–17^ large specific surface area,^18,19^ and high proton conductivity,^16,20–23^ explaining its optimal sensitivity to RH. Indeed, GO can exhibit significant changes in its electrical properties, such as impedance, refractive index, capacitance, and conductivity, in response to variations in environmental humidity.^24–29^ Furthermore, GO can be fully processed in aqueous media or in other harmless and environmentally sustainable organic solvents,^30^ facilitating its thin-film assembly through various deposition techniques on different substrates. Consequently, GO-based humidity sensors can be manufactured on a large scale at a reasonable cost.

The proportions between the OFGs of GO strongly influence its RH detection properties by tuning their interaction with water molecules.^16,20,22,31–41^ Therefore, understanding the relationship between these OFGs and the sensing capabilities of GO is crucial for developing highly sensitive and reliable RH sensors. Research by Fatima et al. demonstrated that GO-based humidity sensors with a higher concentration of −OH groups exhibit better performance than those containing a higher concentration of −C–O–C– groups.^33^ The increased quantity of −OH groups in GO provides more adsorption sites for water molecules, consequently enhancing the protonic conductivity. However, current literature lacks studies examining how other OFGs, such as −C=O, degree of oxidation, and structural defects, affect GO’s humidity detection properties.

Guo et al. demonstrated that laser reduction of GO modifies its degree of oxidation, directly influencing water adsorption/desorption dynamics.^42^ Higher degree of oxidation enhances hydrophilicity but may increase the response time while reducing recovery efficiency. Wee et al. reported that sensors based on GO with ultralarge sheets (47.4 μm) improve proton conductivity along the basal plane due to fewer −COOH groups that block the proton transport pathway.^43^ In a prior study, we reported that GOs synthesized through modifications of Hummers’ method with varying chemical compositions exhibit distinct optoelectronic properties.^44^ The degree of oxidation, flake size, permanent structural defects, and OFG content in GO cannot be individually precisely controlled; therefore, the collective impact of these characteristics allows for tuning their sensitivity to humidity.

In this work, we investigate how the chemical composition (proportion and type of OFGs), degree of oxidation, and the permanent structural defects of GO impact the performance and sensing properties of GO-based humidity sensors. The performance of the devices was systematically tested through resistance and voltage–current curve characterizations over a wide range of relative humidity (11–75% RH) at room temperature (25 °C). Our results indicate significant differences in sensor sensitivity depending on the degree of oxidation, permanent structural defects, and chemical composition of GO samples. Among our three samples, GO-III, which has the highest sp^2^ content, exhibited the lowest sensitivity, whereas GO-II, with higher contents of −OH and −C=O groups, demonstrated the highest performance. Regarding OFGs, a higher content of −OH and −C=O groups might lower the energy barrier for proton hopping. Their highly polar nature favors the formation of strong hydrogen bonding between water molecules and their oxygen atoms, facilitating the water dissociation (H_2_O + H^+^ ↔ H_3_O^+^). This increased the concentration of H^+^, improving the charge transport. This study provides valuable insights into the relationship between the OFGs present in GO and its humidity detection capabilities, particularly highlighting the previously unexplored role of −C=O groups.

## 2 Experimental Section

### 2.1 GO Synthesis

Three types of GO samples with different chemical compositions were synthesized using modifications of Hummers’ method, resulting in GOs with different degrees of oxidation and permanent structural defects. The different OFGs and the degree of oxidation directly influence the sensing properties of GO. Below, the synthesis procedures for each type of GO prepared are detailed. Among the OFGs present in GO samples, −C–O–C– groups are typically found in higher concentrations than −C=O and −COOH acid groups.^45^ It is important to highlight that all synthesis procedures described below are standardized methods with reproducible results already reported in the literature.^44–47^

#### 2.1.1 GO-I Synthesis

GO-I was prepared through a short-time single oxidation step, as detailed by Chen et al.^47^ and Lima et al.^44^ In this procedure, 12 mL of deionized water was slowly added to 46 mL of sulfuric acid (H_2_SO_4_, 98 wt %) with stirring in an ice-water bath to keep the temperature below 10 °C for 15 min. After this, 1.0 g of powder graphite (Synth G1013.06.AH) was added to the mixture, followed by the gradual addition of 3.0 g of potassium permanganate (KMnO_4_) in three portions over 30 min, with a 10 min interval between each addition. The resulting mixture was then transferred to a hot-oil bath and stirred for 2 h at 40 °C. After this time, the dispersion was immersed in an ice-water bath kept at 10 °C, and 300 mL of ice-cold deionized water was gradually added with stirring for 15 min. To stop the oxidation processes, 5 mL of hydrogen peroxide (H_2_O_2_, 30 wt %) was added. The GO-I suspension was purified through three washing cycles with a dilute aqueous solution of hydrochloric acid (DI H_2_O:HCl, 9:1 v/v) using centrifugation, followed by repeated washings with deionized water until the supernatant of the GO suspension reached a pH of around 6–7.

#### 2.1.2 GO-II Synthesis

GO-II was prepared using a long-time oxidation procedure, as described by Lima et al.^44,46^ The process involved two oxidation stages, with the first being a prolonged oxidation step, followed by a second oxidation step that favors the increase in the amount of −C=O groups and induces the breakage of C=C bonds in the basal plane of GO-II.^46^ In the first stage, 5.0 g of graphite flakes (Sigma-Aldrich; 808067) was mixed with 3.75 g of sodium nitrate (NaNO_3_) and 375 mL of sulfuric acid (H_2_SO_4_, 98 wt %) in an ice-water bath. Subsequently, 22.5 g of potassium permanganate (KMnO_4_) was slowly added over 1 h, while the temperature was kept below 5 °C. The mixture was then stirred at room temperature for 120 h, completing the first oxidation stage. In the second stage, 700 mL of a 5 wt % H_2_SO_4_ solution was gradually added over 1 h, and the mixture was heated to 98 °C for 2 h. Finally, the temperature was reduced to 60 °C, and 15 mL of hydrogen peroxide (H_2_O_2_, 30 wt %) was added to conclude the oxidation. The resulting GO-II suspension was purified through repeated washes with H_2_O_2_, H_2_SO_4_, and HCl aqueous solutions, followed by additional washes with deionized water until the supernatant of the GO suspension reached a pH of around 6–7.

#### 2.1.3 GO-III Synthesis

GO-III was also prepared using a single oxidation step reported by Kim et al.^45^ and Lima et al.^44^ Initially, 2 g of powder graphite (Synth G1013.06.AH) was mixed with 45 mL of H_2_SO_4_ (98 wt %) and stirred for 2 h in an ice-water bath. After this, 6.0 g of KMnO_4_ was slowly added to the mixture in three portions over 30 min, with a 10 min interval between each addition. During this stage, the temperature was kept below 10 °C. After all of the KMnO_4_ was added, the mixture was transferred to a hot-oil bath maintained at 35 °C and heated for 2 h. At the end of this time, 200 mL of deionized water was added to the mixture, and the temperature was maintained at around 20 °C for 20 min. The GO-III suspension was purified by three successive washings with a solution of deionized water and HCl using centrifugation, followed by additional washes with deionized water until a pH of around 6–7 was reached.

### 2.2 Fabrication of the Humidity Sensors

To fabricate the sensors, glass substrates (12.5 mm × 25.0 mm) were cleaned with a Piranha solution (H_2_SO_4_:H_2_O_2_) in a 7:3 (v/v) ratio at 80 °C for 30 min. After being rinsed with deionized water, the substrates were stored in isopropyl alcohol (C_3_H_7_OH) for further use. Prior to depositing aluminum (Al) interdigitated electrodes (IDEs) through thermal evaporation in a high vacuum chamber, the substrates were dried with N_2_. The IDEs, as shown in Figure 1a, have an active surface area of 7.0 × 7.0 mm, 120 nm thick, with 0.5 mm wide stripes and a 0.5 mm gap. Next, as illustrated in Figure 1b, 40 μL of a 1.0 mg/mL aqueous dispersion of GO-I, GO-II, or GO-III was dropped onto the IDE area; the substrates were preheated at 60 °C. After drying, the films were then annealed in a vacuum oven at the same temperature for 2 h. Figure 1c shows an illustration of the as-prepared RH sensor featuring a GO film on the Al-IDE surface.

**Figure 1:**
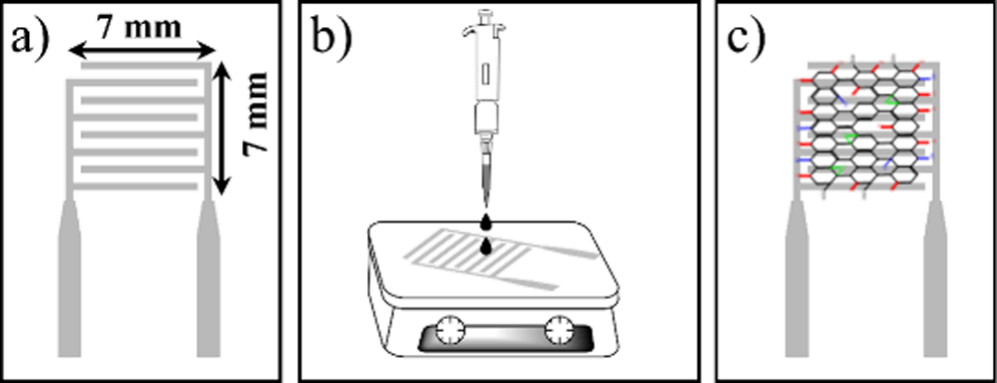
Schematic illustration of GO-based RH sensor preparation. (a) Al interdigitated electrode, (b) GO drop-casting deposition, and (c) as-fabricated GO-based RH sensor.

### 2.3 Characterization and Performance Testing

Transmittance FTIR spectra were collected using a Bruker Vertex 70 instrument equipped with an attenuated total reflectance accessory, operating in the range of 4000 to 400 cm^–1^ with a spectral resolution of 4 cm^–1^. Raman spectroscopy was performed using a Senterra spectrometer from Bruker with a 532 nm excitation wavelength and 2.0 mW laser power at room temperature in a backscattering configuration. X-ray diffraction (XRD) patterns of GO powders were obtained using a Bruker D8 Advance X-ray diffractometer with a Cu Kα radiation source (λ = 1.5406 Å) at 40 keV and a cathode current of 20 mA, over a range of 5° to 80° with a 0.02° resolution. Scanning electron microscopy (SEM) was conducted by using a FEI Quanta 250 microscope at 30 kV. Tapping-mode AFM measurements were made by using a Park System NX10 instrument with a silicon tip at a frequency of 70 kHz. X-ray photoelectron spectroscopy was performed under ultrahigh vacuum medium (Omicron Nanotechnology) using a non-monochromatic Al Kα (*h*ν = 1486.6 eV) X-ray source, with power supplied by an emission of 20 mA at a voltage of 15 kV. The C 1s high-resolution spectra were obtained with an analyzer pass energy of 130 eV and energy steps of 0.025 eV, while the O 1s spectra were acquired with an analyzer pass energy of 130 eV and energy steps of 0.08 eV. Peak fitting was conducted using CasaXPS software, and before the fitting, the background was subtracted using a Shirley function.

The humidity sensing properties of GO-based sensors were investigated at various RH levels at room temperature (25 °C). Different RH levels were achieved using airtight closed glass containers with saturated salt solutions of LiCl, MgCl_2_, K_2_CO_3_, Mg­(NO_3_)_2_, NaBr, CuCl_2_, and NaCl, which produced stable atmospheres with 11, 33, 43, 52, 59, 67, and 75% RH levels, respectively. Electrical measurements were performed using an Ivium Technologies potentiostat/galvanostat (CompactStat model). The device resistance was obtained at 0.1, 0.5, and 1.0 V, with electric currents ranging from −2.0 to 2.0 V. The electrical results that will be presented and discussed later correspond to the behavior of the devices after multiple sensors are manufactured and tested under the same experimental conditions.

## 3 Results and Discussion

### 3.1 Spectroscopic and Structural Characterizations of GOs

The FTIR and Raman spectra provide insights into the structural and chemical characteristics of the GO-I, GO-II, and GO-III samples and were discussed in more detail in the Supporting Information. As shown in Figure S1a, the FTIR transmittance spectra reveal characteristic vibrations of various OFGs, including broad O–H and C–H stretching bands (3700–2600 cm^–1^), a −C=O stretching band at 1720 cm^–1^, as well as signals associated with water absorption, C=C stretching in nonoxidized carbon (particularly prominent in GO-III), and C–OH and C–O vibrations.^48,49^ These spectral features indicate a higher sp^2^ carbon content in GO-III compared with the other samples. Figure S1b presents the Raman spectra, in which all samples exhibit the characteristic D and G bands of graphene oxide, along with second-order bands (2D, D+G, D+D′). The low intensity of the 2D band and the D/G intensity ratio suggest a high density of structural defects and a disrupted stacking order, with all samples showing similar defect levels.^50,51^ The differences between the samples lie in the sp^2^/sp^3^ carbon ratio and in the percentage of each type of OFG, as it will be discussed below.

To further investigate the degree of oxidation and chemical composition of GO samples, X-ray photoelectron spectroscopy (XPS) analysis was performed. Figure 2a presents the XPS survey spectra of GOs, in which all samples exhibit two characteristic peaks centered at 284.6 and 531 eV, corresponding to the C 1s and O 1s, respectively.^52,53^ The atomic percentages were determined based on the relative intensities of these survey spectra. From the XPS wide-scan spectra, the carbon and oxygen contents in GO-I were found to be 70.26% and 29.74%, while in GO-II and GO-III, they were 71.72 and 28.28%, and 73.30 and 26.70%, respectively. The calculated C/O ratios for GO-I, GO-II, and GO-III were 2.36, 2.54, and 2.75, respectively, indicating a significant degree of oxidation of GO samples.^45,47,54^ These values indicate that GO-I presents the highest level of oxidation, GO-II has intermediate oxidation, and GO-III is the least oxidized.

**Figure 2:**
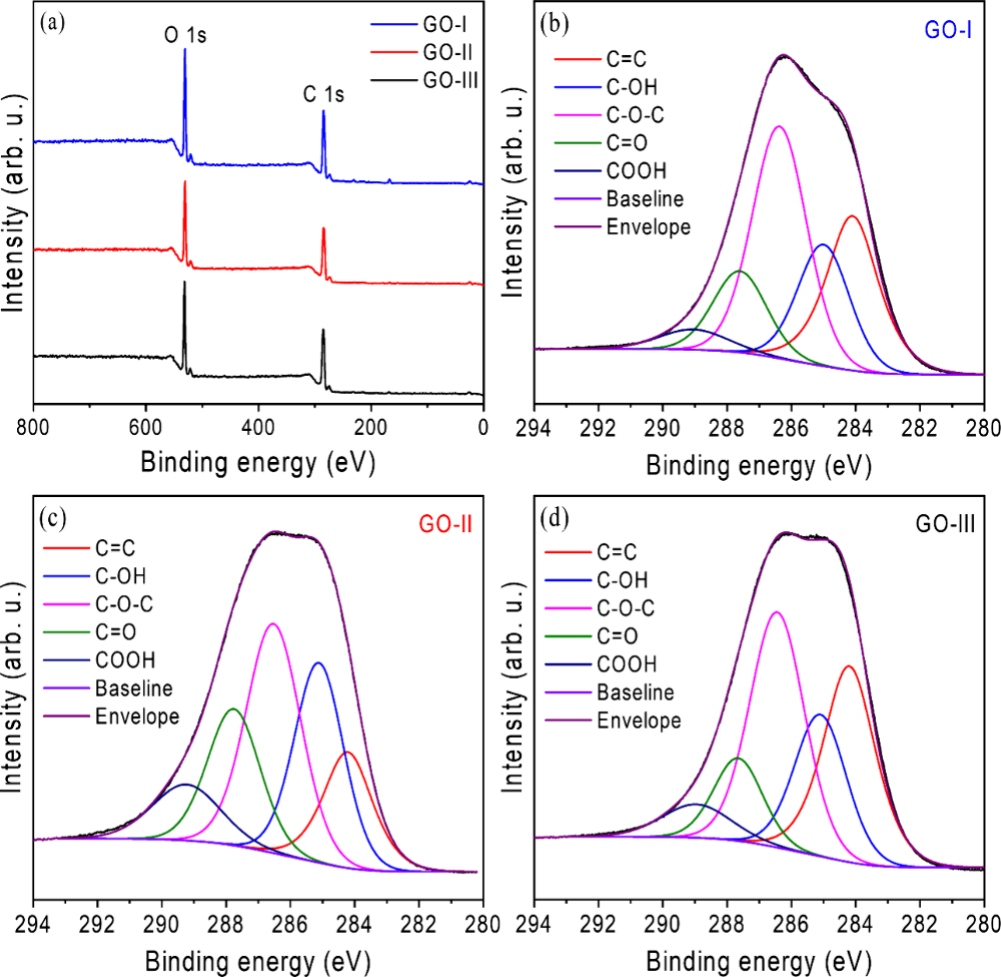
(a) XPS survey spectra of GO-I, GO-II, and GO-III, showing characteristic peaks corresponding to the carbon (C 1s) and oxygen (O 1s) bonding states and (b–d) deconvoluted XPS spectra of C 1s of GO-I, GO-II, and GO-III, respectively.

Figure 2b–d presents the high-resolution C 1s XPS spectra of the three GO samples, in which the deconvoluted bands and their respective calculated relative areas (%) provide a detailed evaluation of their chemical composition. All spectra display a characteristic peak at 284.2 eV, attributed to unoxidized carbon atoms (C=C), representing the intact sp^2^-hybridized graphene lattice. Additionally, four distinct peaks at higher binding energy are observed at 285.1, 286.4, 287.7, and 289.0 eV, corresponding to –OH, –C–O–C–, −C=O, and −COOH functional groups, respectively.^53,55,56^ Table 1 summarizes the quantitative analysis of each chemical group within the GO samples.

**Table 1:** Elemental Composition and Relative Amounts of Functional Groups (in at %) of Samples Evaluated by XPS Analysis

sample	elemental composition (%)			peak area ratio (%)				
	C	O	C/O ratio	C=C	–**OH**	–**C**–**O**–**C**–	–**C=O**	–**COOH**
GO-I	70.26	29.74	2.36	26.16	18.14	38.26	13.20	4.25
GO-II	71.72	28.28	2.54	15.12	25.00	31.85	18.29	9.73
GO-III	73.30	26.70	2.75	28.56	19.82	34.61	10.87	6.15

One observes that a lower C=C peak intensity was found for GO-II, indicating the lowest sp^2^ carbon content. As previously discussed in our study, the degree of oxidation is directly influenced by the oxidizing agents used during GO synthesis, and GO-II was prepared with two sequential oxidation steps that favor the formation of a greater number of −C=O groups, explaining its lower sp^2^ carbon content and greater amount of permanent structural defects.^44^ All techniques—XPS, FTIR, and Raman—corroborate each other, consistently indicating GO-I as the most oxidized, GO-II with moderate oxidation and a greater amount of permanent structural defects, and GO-III with the lowest oxidation.

X-ray diffraction (XRD) characterizations were also carried out to investigate the crystalline nature of the samples in greater detail, such as the distance between layers in the GO films.^45^ For comparison purposes, the XRD patterns of pure graphite flakes and powder are depicted in Figure 3a. In both cases, an intense and sharp peak at 2θ = 26.5° is observed, corresponding to the diffraction plane (002) with an interlayer distance (ID) of 3.36Å.^45,57^ The diffraction patterns of the samples are shown in Figure 3b. The absence of a diffraction peak at 26.5° in the diffractograms indicates complete oxidation of graphite. The presence of distinct oxygen-containing functional groups induces the formation of new peaks at lower diffraction angles, which are associated with the (002) plane.^57^ The XRD patterns of GO-I, GO-II, and GO-III showed broad peaks centered around 2θ = 10.4°, 2θ = 10.8°, and 2θ = 10.0°, corresponding to IDs of 0.850, 0.818, and 0.884 nm, respectively. These distances suggest that the oxidative method used results in highly oxidized and exfoliated GO sheets, mainly composed of uncoupled layers.^46^

**Figure 3:**
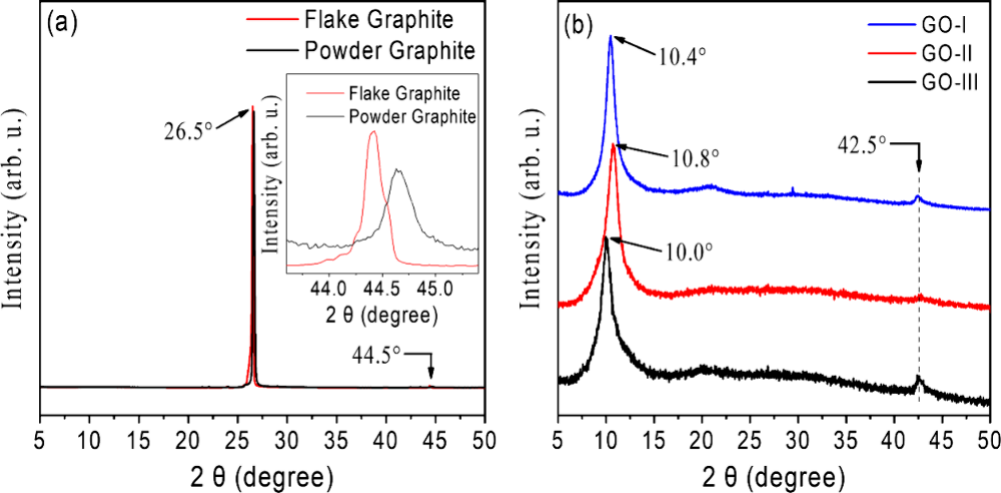
X-ray diffraction pattern of (a) flake and powder graphite precursors and (b) as-prepared GO-I, GO-II, and GO-III.

When analyzing the differences in the ID obtained, based only on the degree of oxidation, we obtain a distinct trend from the literature, in which a higher degree of oxidation causes an increase in the ID.^58^ However, the interlayer distance in hydrated GO is also strongly influenced by the nature and amount of OFGs, particularly through hydrogen bonds with intercalated water molecules.^44,59^ Groups such as −OH, −C–O–C–, −C=O, and −COOH contribute differently to these interactions, depending on their polarity and spatial orientation. A higher concentration of C=O and −OH groups tends to reduce the ID in graphene oxide, while −C–O–C– groups contribute to its increase.^59–61^ Among them, −C=O seems to exert the greatest influence on the reduction of the spacing, possibly due to its more polar character. This trend is evident in the GO-II sample, which presents the highest proportion of polar groups and the smallest ID. Nevertheless, further investigation is needed since the available literature does not provide sufficient data on how each OFG and its correlation with degree of oxidation impact the ID of GO.

As proposed by Karim et al., the poor interlayer interaction in GO, resulting from its large interlayer distance, enhances proton conductivity.^16,62^ Furthermore, a large ID can also provide the device a fast response.^63,64^ Based solely on this information, one might expect that GO-III would exhibit the best proton conductivity among the three GOs studied, given the higher interlayer distance for this sample. However, proton conductivity also depends on the degree of oxidation and the types of oxygenated groups present.^16,20,22,31,34,36^ Thus, relying solely on the ID value can be misleading, as distinct OFGs affect the ID differently. For instance, significant concentrations of −C=O groups tend to reduce the ID value, whereas high levels of −C–O–C– groups increase it. Additionally, as will be illustrated later in the section 3.2, the −C–O–C– groups contribute less to proton conductivity compared to –OH groups.

The diffraction patterns of GO also show another low-intensity peak around 42.5°, associated with the 100 crystalline plane, as observed in Figure 3b. This peak allows for an estimation of the distance between equivalent carbon atoms in the hexagonal lattice, which is ideally 2.46 Å for graphene.^65^ For GO, it was estimated at approximately 2.12 Å, indicating that the hexagonal structures are distorted due to structural defects such as holes and vacancies. Therefore, GO-II seems to have the most permanent structural defects among the samples, showing the lowest peak intensity at 42.5°. These findings align well with the observations from the XPS, FTIR, and Raman analyses, reinforcing the conclusion that GO-I is the most oxidized sample, followed by GO-II, with GO-III being the least oxidized. This is also consistent with the performance of the humidity sensors, as discussed in the following section.

The morphology of the GO films deposited on the IDEs was analyzed using a scanning electron microscopy (SEM) technique. As shown in Figure 4, the GO-I, GO-II, and GO-III films exhibit uniform coverage and a wrinkled morphology typical of the GO layers. In addition, the atomic force microscopy (AFM) data shown in Figure S2 revealed distinct differences in surface roughness among the GO samples. The RMS roughness values were 68 ± 5, 39 ± 7, and (12 ± 2) × 10 nm for the GO-I, GO-II, and GO-III films, respectively. The lower roughness observed for GO-II may be associated with smaller flake sizes and better dispersion, resulting in more homogeneous films. This smoother morphology could contribute to more uniform adsorption of water molecules and enhanced proton transfer efficiency, whereas the higher roughness in GO-III may disrupt proton conduction pathways and interfere with proton transfer via the Grotthuss mechanism, thereby reducing sensing performance. These differences in surface morphology may therefore partially account for the variation in sensor sensitivity among the samples. The extent of area coverage was further confirmed by X-ray diffraction. The wrinkled appearance of the GO sheets results from interactions between adjacent layers as multiple GO sheets stack during film formation. Shen et al. reported that, during the thin-film formation, GO sheets fold due to the surface tension of water in the drying process.^66^ This pronounced roughness, along with the high porosity of the films, provides a large surface area, enhancing the adsorption and desorption of water molecules. These morphological and chemical characteristics make GO highly hydrophilic and electrically insulating.^46,67^

**Figure 4:**
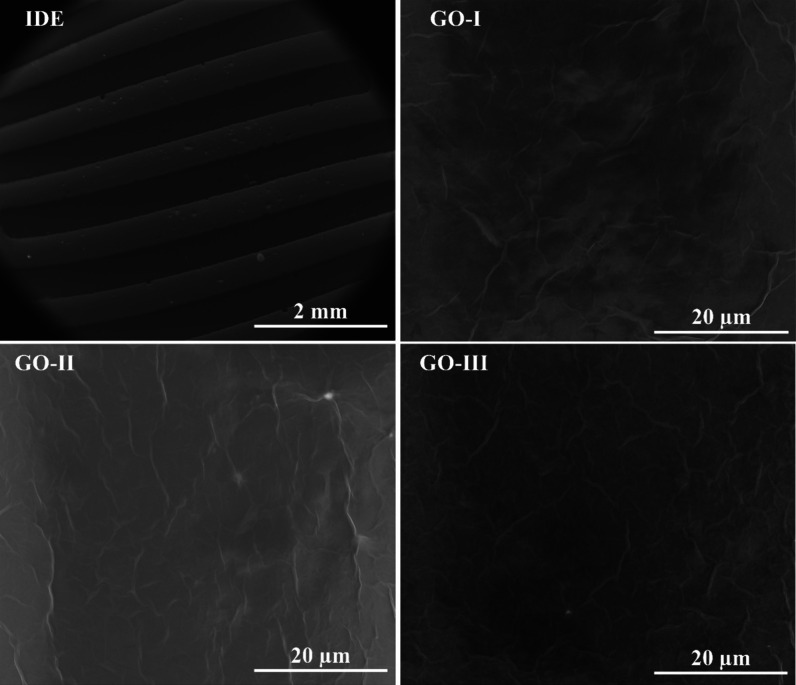
SEM images of the GO-I, GO-II, and GO-III surfaces deposited onto the IDE. One observes wrinkles typical of GO films.

### 3.2 Humidity Sensing Characterization

Figure 5a–c shows the current–voltage (*I*–*V*) characteristics of the humidity sensors based on the three GO films, measured in the range −2.0 to 2.0 V under RH levels from 11 to 75% at room temperature. The results indicate that, under identical bias voltage conditions, the current magnitude increases with rising relative humidity. For instance, applying a bias voltage as low as +0.1 V is sufficient to distinguish different humidity levels. At + 0.1 V, the electrical currents for GO-I, GO-II, and GO-III increase drastically from 0.083 ± 0.009, 0.064 ± 0.003, and 0.84 ± 0.03 to 84.6 ± 0.4, 87 ± 1, and 209.4 ± 0.3 nA, respectively. This trend suggests that GO films predominantly exhibit *n-*type semiconductor behavior, as they accept electrons into their valence band when exposed to *n-*dopant analytes, such as H_2_O. The current increase is interpreted as a reduction in the energy gap between the Fermi level and the valence band, together with a decrease in hole carrier concentration.^68^

**Figure 5:**
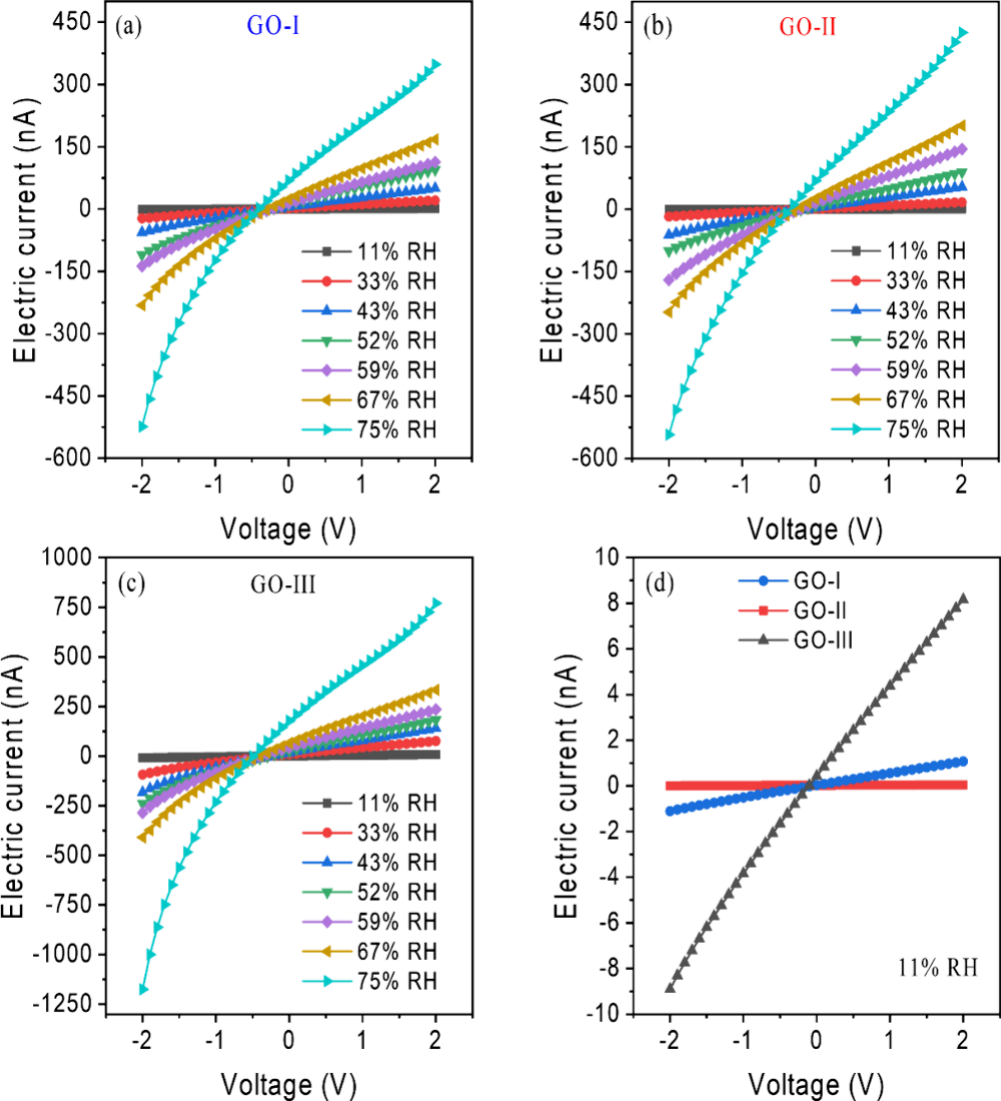
Current–voltage (*I*–*V*) characteristics of GO-based humidity sensors in a RH range from 11 to 75% of (a) GO-I, (b) GO-II, (c) GO-III, and (d) ohmic region of GOs under 11% RH.

The electrical nature of GO (*n-* or *p-*type) is primarily determined by the presence of OFGs, which can withdraw or donate electrons to the GO backbone through resonance or inductive effects. For example, the findings of Tu et al. show that −C=O, –COOH, sp^3^-bonded −OH, and −C–O–C– groups act as electron donors, while sp^2^-bonded −C–O–C– and –OH groups act as electron acceptors in the interaction with water molecules.^69^ Chen et al. also highlighted the more pronounced electron-donating effect of sp^2^-bonded −OH groups compared to the electron-withdrawing effect.^70^ However, it is widely accepted in the literature that the overall electronic properties of GO are influenced by the combined effects of all functional groups present in GO films. Films that exhibit a reduction in electrical resistance when exposed to RH are classified as *n-*type, whereas those that exhibit an increase in electrical resistance are *p-*type.^32,33,69,71^ Based on our experimental results, all GOs demonstrated *n*-type behavior. This result is further supported by the C 1s XPS analysis, which revealed that all samples exhibited a higher concentration of electron-donor groups (−OH and −C–O–C−) compared to electron-withdrawing groups (−OH and −COOH). Among them, GO-II had the highest proportion of −OH and −C=O groups, which are known to enhance the interaction with water molecules, facilitating charge transport and improving sensor performance.

Within the voltage range of −0.5 to 0.5 V, the *I*–*V* curves of GO samples exhibit a linear behavior at all humidity levels, indicating the formation of an ohmic contact at the interface between aluminum (Al) and GO.^72,73^ Figure 5d illustrates this behavior specifically for GO-I, GO-II, and GO-III at an RH of 11%. The presence of an ohmic contact facilitates the direct injection of electrons at the interface between the electrode and the GO material.^74^ Consequently, the sensor response primarily originates from the GO sensing layer, simplifying the analysis of the results.^33^ Furthermore, as can be seen in Figure 5a–c, at voltages greater than 0.5 V, the *I*–*V* curves exhibit behavior analogous to that of a Schottky barrier, which becomes more pronounced with increasing RH. The applied electric field can cause the ionization of absorbed water molecules (H_2_O → H^+^ + OH^–^), a phenomenon strongly dependent on RH, applied voltage, sensing layer thickness, and width of the IDE gap.^75,76^ The ohmic contact between the Al-GO junction results from the donation of H^+^ from ionized water molecules to the GO, reducing its resistance and leading to the partial reduction of GO (GO + H^+^ + e^–^ = *r*GO + H_2_O).^75–77^

When water molecules interact with GO, both adsorption and intercalation occur. Figure 6 illustrates the sensing mechanism for GO-based sensors, in which OFGs, such as −OH, −C=O, and −C–O–C–, provide active sites for water interaction.^22,78^ At low RH, water molecules adsorb physically, self-dissociate into −OH radicals, and remain restricted due to strong double hydrogen bonds with −OH groups on GO.^79,80^ The presence of −C=O groups in the basal plane and at the edges of GO, with their highly polar character, favors the presence of permanent structural defects (carbon vacancies) and consequently increases the adsorption and migration of water molecules to the internal layers of GO.

**Figure 6:**
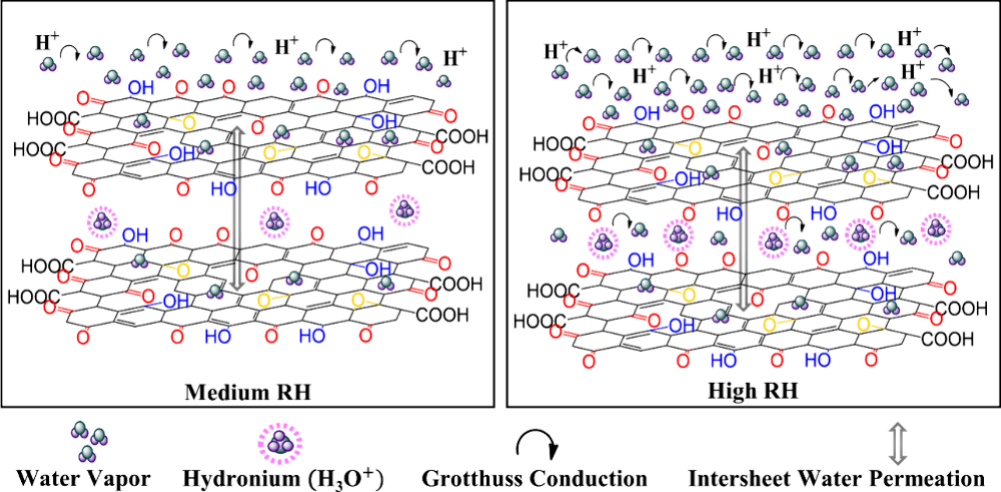
Sensing mechanism for GO-based sensors. At medium RH, conductivity is mediated mainly by the Grotthuss mechanism. At high RH, water penetrates deeper into the GO layers, allowing H^+^ jumping and the H_3_O^+^ ions to diffuse.

As RH increases, additional water molecules adsorb via hydrogen bonding, facilitated by low energy barriers (0.016 eV for adjacent −OH, 0.12–0.14 eV for nonadjacent ones).^22^ However, proton hopping between −C–O–C– groups is limited due to higher energy barriers (0.21 eV for adjacent −C–O–C–, 0.42 eV for nonadjacent −C–O–C−).^22^ Through the hydrogen-bonding network, protons can hop between the −OH and −C–O–C– functional groups. The −OH groups in GO interact with adsorbed water, releasing protons (H^+^) into the aqueous environment, which leads to the formation of hydronium ions (H_3_O^+^) through the equilibrium reaction (H_2_O + H^+^ ↔ H_3_O^+^). These H_3_O^+^ ions then donate a proton to an adjacent water molecule, forming new H_3_O^+^ ions in a continuous chain reaction. This proton transport mechanism is known as the Grotthuss chain reaction.^81^

At high RH, water permeates GO layers,^82^ promoting the hydrolysis of functional groups (−COOH, −OH).^83^ This process generates additional H_3_O^+^ ions, enhancing proton transfer and ionic conductivity, in which H^+^ becomes the dominant charge carrier.^84^ Additionally, hydrolysis increases the interlayer spacing in GO, weakening hydrogen-bonding interactions and creating water channels that facilitate proton transport.^21,85^

The effect of humidity on conductivity is reflected in the electrical resistance of GO films, providing insight into their sensing performance. Figure S3a–c shows the logarithm of the electrical resistance as a function of RH at voltages of 0.1, 0.5, and 1.0 V. Three specific data points were selected from the plot to evaluate the overall sensitivity behavior. As can be observed in Figure 7a, at 0.1 V, the electrical resistance values of GO-I, GO-II, and GO-III were (121 ± 3) × 10^7^, (156 ± 8) × 10^7^, and (119 ± 6) × 10^6^ Ω at 11% RH, which changed to (118 ± 1) × 10^4^, (115 ± 3) × 10^4^, and (478 ± 6) × 10^3^ Ω at 75% RH, respectively. The linear regression of the logarithmic sensor’s resistance data shows good linearity with *R* ^2^ > 0.95 for all devices. To evaluate and compare the performance of these humidity sensors both among themselves and with existing devices in the literature, the sensitivity (*S*) was determined by

$$S\%=\left(\frac{G_{\mathrm{high}}-G_{\mathrm{low}}}{G_{\mathrm{low}}\times \Delta \mathrm{RH}}\right)\times 100\%$$ 1

where *G*_high_ is the conductance at the highest RH level, *G*_low_ is the reference conductance at the lowest RH level, and ΔRH is the variation between the maximum and minimum RH level (here, a range of 11–75% RH was used, yielding ΔRH = 64%). This equation applies when the resistance decreases with increasing humidity. If the sensors exhibit an increase in resistance with increasing RH, the conductance is replaced by the resistance (*R*) in eq 1.^29,86^

**Figure 7:**
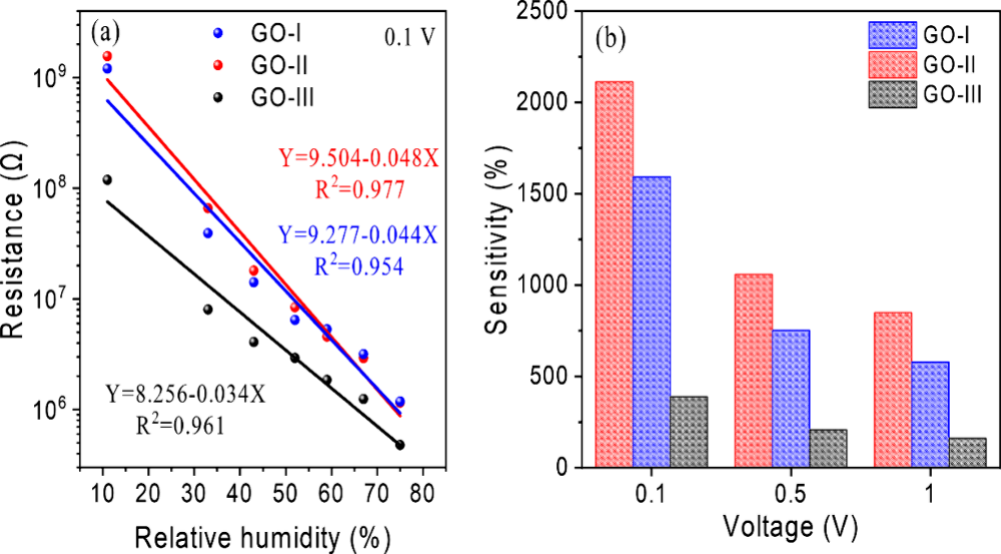
(a) Logarithm of the electrical resistance of GO samples as a function of RH at 0.1 V and (b) sensitivities for DDP of 0.1, 0.5, and 1.0 V.

The sensitivities obtained are shown in Figure 7b; they decrease with an increasing applied voltage for all sensors. At 0.1 V, GO-I, GO-II, and GO-III exhibited sensitivity values of 1592 ± 1, 2113 ± 2, and 388.1 ± 0.4%, respectively. This decrease in sensor response with increasing voltage may be attributed to a reversible reduction in GO, as reported in other studies.^75,76,87^ Table 2 summarizes the performance of our sensors and compares them with those of other GO-based sensors reported in the literature. One can observe that the sensitivity of the GO-II sensor in this work exceeds that of GO-I and GO-III. Furthermore, our GO-II device operating at low voltage exhibits a sensitivity comparable to that of untreated GO-based sensors, which ranges from 56% to 1362%.

**Table 2:**
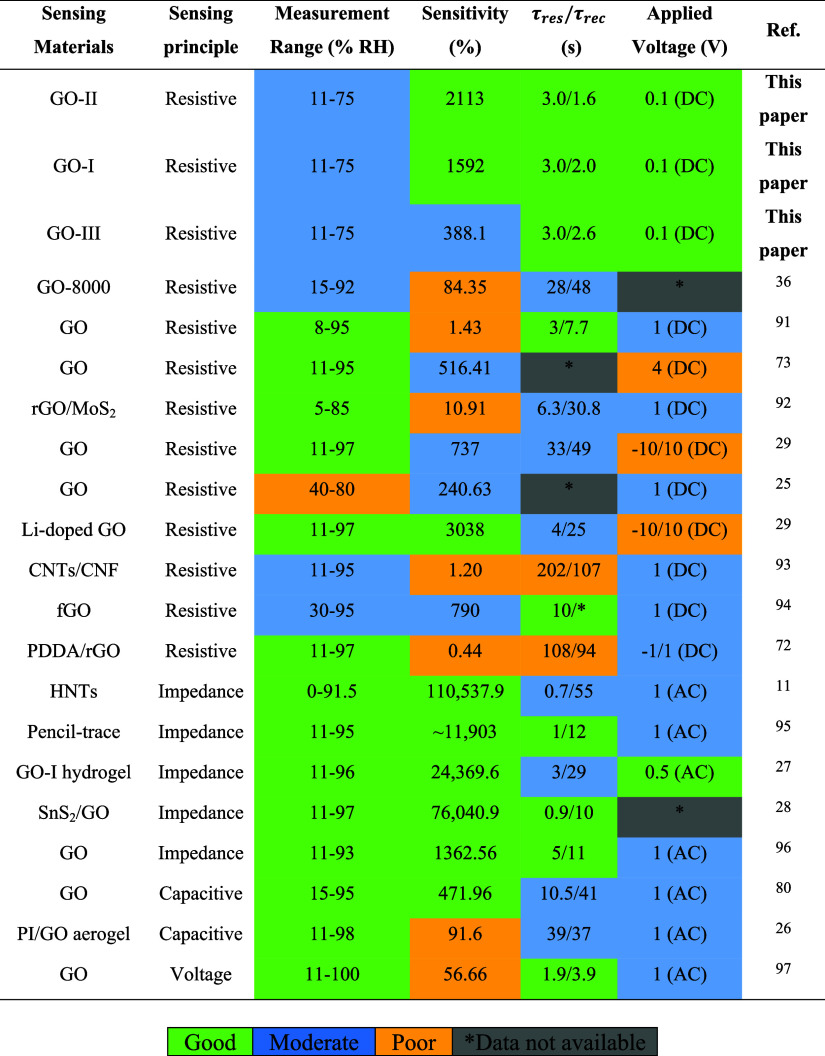
Humidity Sensors Reported in the Literature for Different Sensing Materials

Among the three sensors, the GO-II-based device exhibited a superior performance in terms of humidity sensitivity and responsiveness. This improved behavior can be attributed to the optimized chemical composition and structural characteristics of GO-II. XPS analysis revealed that GO-II possesses the lowest sp^2^ carbon content and the highest combined proportion of −C=O and −OH groups, both of which are highly polar and play a crucial role in water molecule adsorption, proton dissociation, and conduction via the Grotthuss mechanism.^16,21,43,63^ Notably, C=O groups exhibit even higher polarity than −OH, contributing significantly to water affinity.^78,88^ Furthermore, the synthesis of GO-II involved two oxidation steps, resulting in an oxidized material with numerous permanent structural defects (e.g., carbon vacancies and ripples). The presence of pores and exposed sheet edges facilitates the diffusion of water molecules into the interlayers of GO-II, promoting an increase in proton hopping due to the increased number of ion exchange sites.^31,37,64^ Despite presenting a slightly lower overall degree of oxidation than GO-I (as indicated by its C/O ratio), the specific distribution and abundance of these functional groups make GO-II particularly effective for moisture sensing. Furthermore, its smaller interlayer spacing (0.818 nm), resulting from the high density of oxygenated polar groups, facilitates a stronger interaction with water molecules and enhances the ion conduction pathways. In addition to chemical composition, morphological features also play a role in humidity sensing. A smoother GO surface, with a higher density of sheets, may promote the formation of a more continuous water layer, facilitating proton hopping and reducing scattering or trapping sites that hinder charge transport. Conversely, increased surface roughness, as observed for GO-III, can be advantageous due to the larger interlayer spacing; however, it may also introduce irregularities that disrupt uniform water adsorption—such as extensive empty spaces—thereby limiting efficient conduction. Raman analysis also indicates a high degree of structural disorder in GO-II, which further corroborates the increased water adsorption and charge transport. Collectively, these factors explain the superior sensing performance of the GO-II-based sensor.

To complete the sensor characterization, hysteresis, response time, and recovery time were studied to evaluate the stability and performance of the humidity sensors. The hysteresis was measured by varying RH from 11 to 75% (absorption) and reducing it from 75 to 11% (desorption), as shown in Figure 8a–c. The maximum hysteresis values of GO-I, GO-II, and GO-III were determined to be 53.04, 31.65, and 37.50% under 11% RH, respectively. At RH greater than 11%, the hysteresis was less than 4% for all samples, indicating that the adsorbed water is difficult to release from the inner layers of the GO films, especially at low RH. High hysteresis can also be associated with high sensor sensitivities, as there is a direct correlation between these two properties, in which increased sensitivity results in a device with greater hysteresis.^89^ Furthermore, as suggested by the work of Zhang et al.,^72,90^ exposing the sensor to dry air, conditioned with phosphorus pentoxide (P_2_O_5_) powder (RH 0%), between two humidity measurements, favors the recovery of the device through the removal of adsorbed water molecules. This procedure can contribute to the minimization of the hysteresis effects.

**Figure 8:**
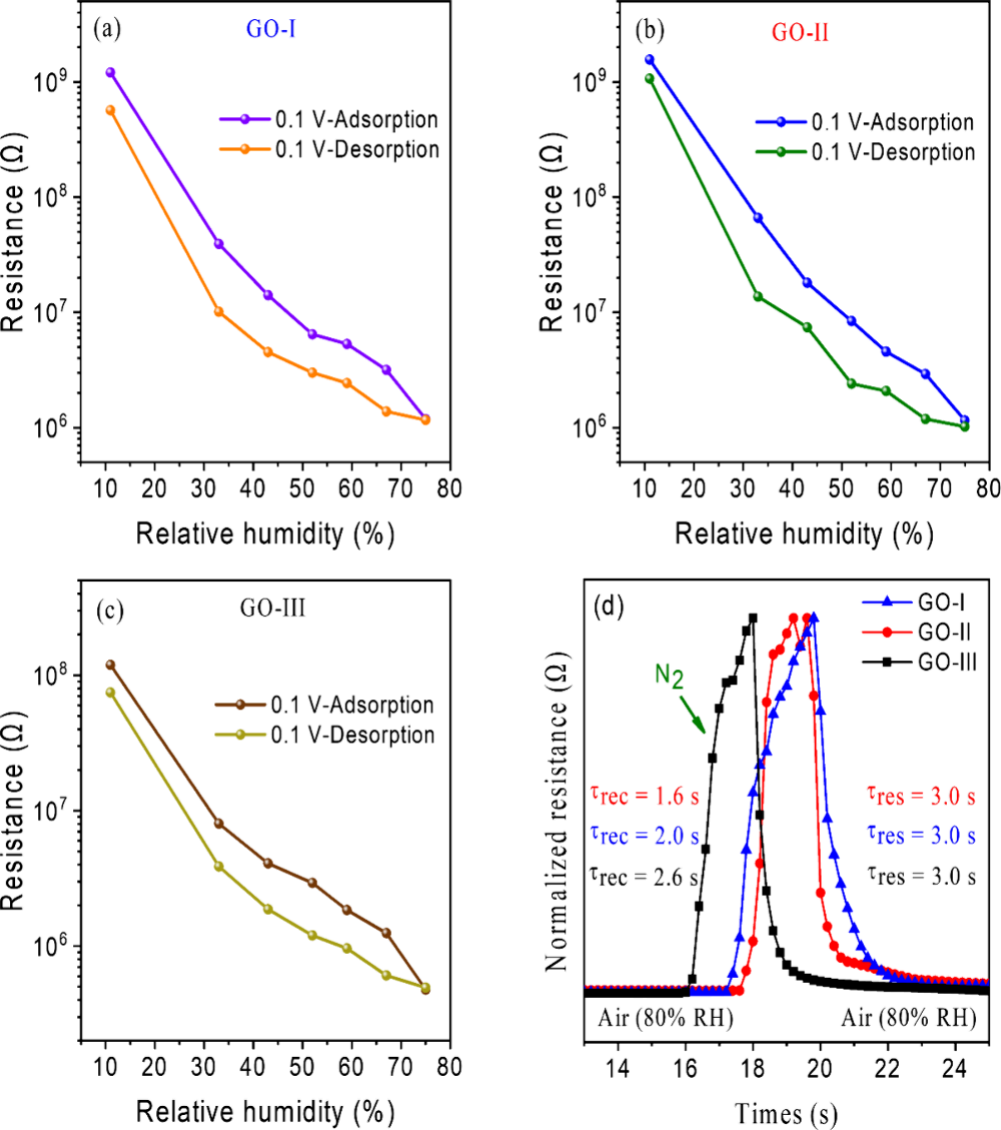
Hysteresis curve of adsorption–desorption of (a) GO-I, (b) GO-II, and (c) GO-III and (d) response and recovery times of the sensors subjected to an abrupt change in the RH.

The dynamic responses of the devices were evaluated by subjecting them to a rapid change in humidity, ranging from the RH of ambient air (80% RH) to a low RH level achieved by a short stream of N_2_. In Figure 8d, the sensors’ response to this RH change is shown in two steps: response time (τ_res_) and recovery time (τ_rec_). GO-I, GO-II, and GO-III exhibited response times of 3.0 s and recovery times of 2.0, 1.6, and 2.6 s, respectively. This swift performance is attributed to the hydrophilic OFGs, the large interlayer distance (>0.818 nm), and the numerous defects in the GOs. The quick response aligns with a modeling study by Wei et al., which emphasized the ultrafast water transport in GO membranes due to their porous microstructures.^37^ Compared with the humidity sensors presented in Table 2, our GO-based humidity sensors exhibit high sensitivity and response and recovery times that are as fast as those shown in previous reports, highlighting the potential of GOs for real-time RH monitoring.^91–97^

## 4 Conclusions

In summary, we fabricated GO-based humidity sensors with varying chemical compositions, degrees of oxidation, and permanent structural defects and carefully investigated how these characteristics influence their performance. Comprehensive analyses, including FTIR, XPS, Raman spectroscopy, AFM, and XRD, confirmed the highly oxidized nature and defective structure of our GO samples, making them well-suited for humidity sensing applications. The sensors demonstrated significant sensitivity variations depending on their degree of oxidation, permanent structural defects, and chemical composition. GO-II, characterized by a balanced degree of oxidation and the highest concentration of −OH and −C=O groups, outperformed this work in terms of sensitivity. This highlights the crucial role of functional group distribution and permanent structural defects, rather than the degree of oxidation alone, in determining sensor performance.

Notably, GO-II exhibited superior sensitivity due to its high concentration of hydrophilic −OH groups, which facilitate water adsorption, and its low C–O–C– content, which reduces the energy barrier for proton hopping. Furthermore, the presence of −C=O groups that favor the formation of permanent structural defects, together with their highly polar nature, indicates an increase in the adsorption and migration of water molecules' adsorption and migration into the internal layers of GO. This mechanism results in an increase in proton conduction and sensor sensitivity. Additionally, the moderate surface roughness observed in GO-II may have contributed to enhanced water molecule interaction and diffusion, further supporting its superior performance.

The GO-II-based humidity sensor demonstrated effective performance, being capable of detecting a broad humidity range of 11–75% RH at a minimum applied voltage of 0.1 V. Its sensitivity reached an exceptional 2113%, accompanied by rapid response and recovery times of 3.0 and 1.6 s, respectively. The maximum hysteresis value observed was 31.65% at 11% RH. These findings offer important perspectives on the influence of the degree of oxidation, permanent structural defects, and the OFGs on humidity sensing, emphasizing a critical and underexplored aspect of GO-based sensors. Our work contributes to the development of sensitive and fast-response GO-based humidity sensors, highlighting GO-II due to its favorable combination of OFGs, degree of oxidation, and permanent structural defects as a potential material for real-time humidity sensing applications.

## Supplementary Material



## Notes

(a) Typical FTIR spectra and (b) Raman spectra highlighting the D and G bands, along with the 2D, D+G, and D+D’ bands of GO-I, GO-II, and GO-III films (Figure S1); AFM topographic and 3D surface profile images of GO-I, GO-II, and GO-III films, acquired over a scan area of 30 μm^2^ (Figure S2); and logarithm of resistance as a function of RH of sensors based on (a) GO-I, (b) GO-II, and (c) GO-III, for 0.1, 0.5, and 1.0 V (Figure S3) (PDF)
