# Supplementary material for: Impact of GO Chemical Composition on the Performance of Humidity Sensors
Source: ACS Omega. 2025 Jul 18;10(29):32257–68. doi: 10.1021/acsomega.5c04175 (PMC12311742; doi:10.1021/acsomega.5c04175)
Supplement: Supplementary file 1 [file ao5c04175_si_001.pdf]

Supporting Information

Impact of GO Chemical Composition on the Performance of Humidity Sensors

Nayton C. Vicentini[1], Alessandro H. Lima[1], Giovanni R. Carvalho[1], Camila T. Tavares[1], Anne C. P. Fernandes[1], Clemilda C. S. Cunha[1], Joyce R. Araújo[2], Sanair M. S. Palheta[2], Benjamin Fragneaud[1], Indhira O. Maciel[1], Cristiano Legnani[1] and Welber G. Quirino[1*].*

[1]Nanoscience and Nanotechnology Group - Nano, Physics Department, UFJF, Juiz de Fora-MG, 36036-900, Brazil.
[2]Materials Metrology Division, National Institute of Metrology, Quality and Technology - INMETRO, Duque de Caxias-RJ, 25250-020, Brazil.

[*]Corresponding authors
E-mail address: naytonvicentini@hotmail.com; wgquirino@ufjf.br

1. Infrared and Raman Spectroscopy

Figure S1a presents the transmittance FTIR spectra of GO-I, GO-II, and GO-III, revealing vibrations characteristics of several OFGs. A broad and intense absorption band is observed in the range of 3700-2600 cm^{-1}, which corresponds to O−H and C−H stretching vibrations, as well as the presence of adsorbed water molecules in the samples.[1–3] This broad band is a characteristic signal commonly found in the FTIR spectra of GO samples.[4–8] Additionally, a narrow band at 1720 cm^{-1} is attributed to the −C=O stretching vibrations of −C=O and −COOH acid groups.[9,10] The band at 1600 cm^{-1}, related to water absorption, is more prominent in the GO-I and GO-II samples, where the characteristic band at 3200 cm^{-1} is also intensified.[11,12] The band at 1581 cm^{-1}, observed primarily in the GO-III sample, corresponds to the C=C stretching mode of non-oxidized carbon.[9,10] Although this band is more pronounced in GO-III, it is also superimposed with the band at 1620 cm^{-1} in GO-I and GO-II, suggesting that GO-III has the highest sp^2 carbon content among the samples. Deformation and stretching frequencies of C−OH bonds, associated with −OH groups, are confirmed by absorption bands at 1370 cm^{-1} and 1410 cm^{-1}.[2,13] The bending vibrations of the C−O−H fragment in −COOH groups appear at 1247 cm^{-1} and 1172 cm^{-1}. Finally, the bands located at approximately 1040 cm^{-1} and 980 cm^{-1} are attributed to C−O vibrations in −OH and −C−O−C− groups.[14,15]

Raman spectroscopy measurements are shown in Figure S1b. All samples exhibit two broad and intense bands typical of GO: the D and G bands, centered around 1348 cm^{-}

[1] and 1593 cm^{-1}, respectively.[16,17] The G band is related to in-plane stretching of the C-C bonds, while the D band is defect-activated and related to the breathing mode of the hexagonal carbon ring.[16,18,19] The Raman spectra also exhibit additional low-intensity second-order bands around 2700, 2920, and 3170 cm^{-1}, corresponding to the 2D, D+G, and D+D' bands, respectively.[16] The low intensity of the 2D band, together with the intensity ratio between the D and G bands, indicates a high defect concentration and the breaking of the stacking order, as expected for GO. As Raman spectroscopy with a 532 nm excitation wavelength is sensitive only to sp^2 carbon, it can be observed that the aromatic clusters in all samples have similar sizes, with the same amount of defects.[19,20]

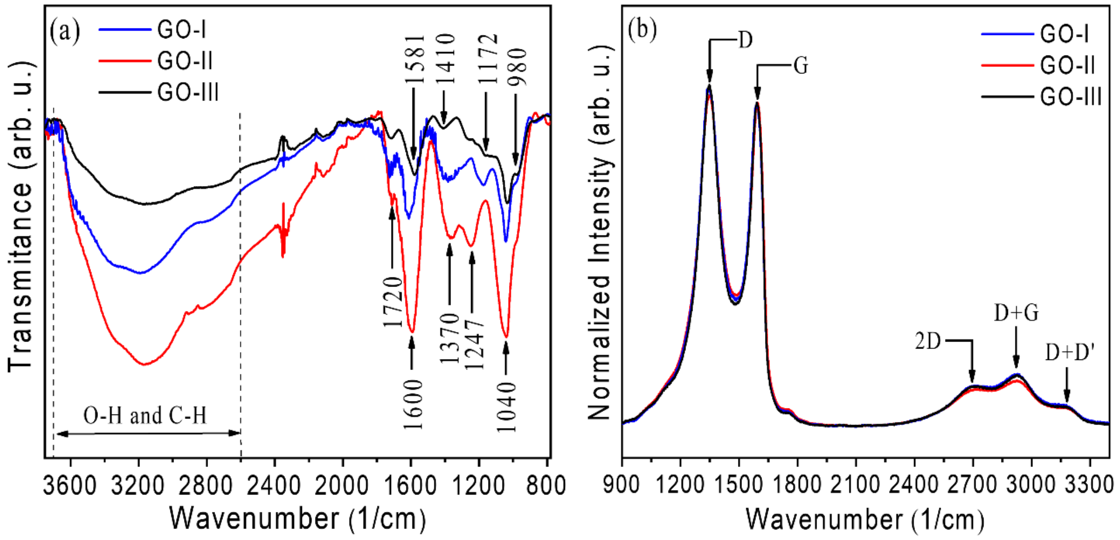

Figure S1: (a) Typical FTIR spectra and (b) Raman spectra highlighting the D and G bands, along with the 2D, D+G, and D+D' bands of GO-I, GO-II, and GO-III films.

2. Supporting figures

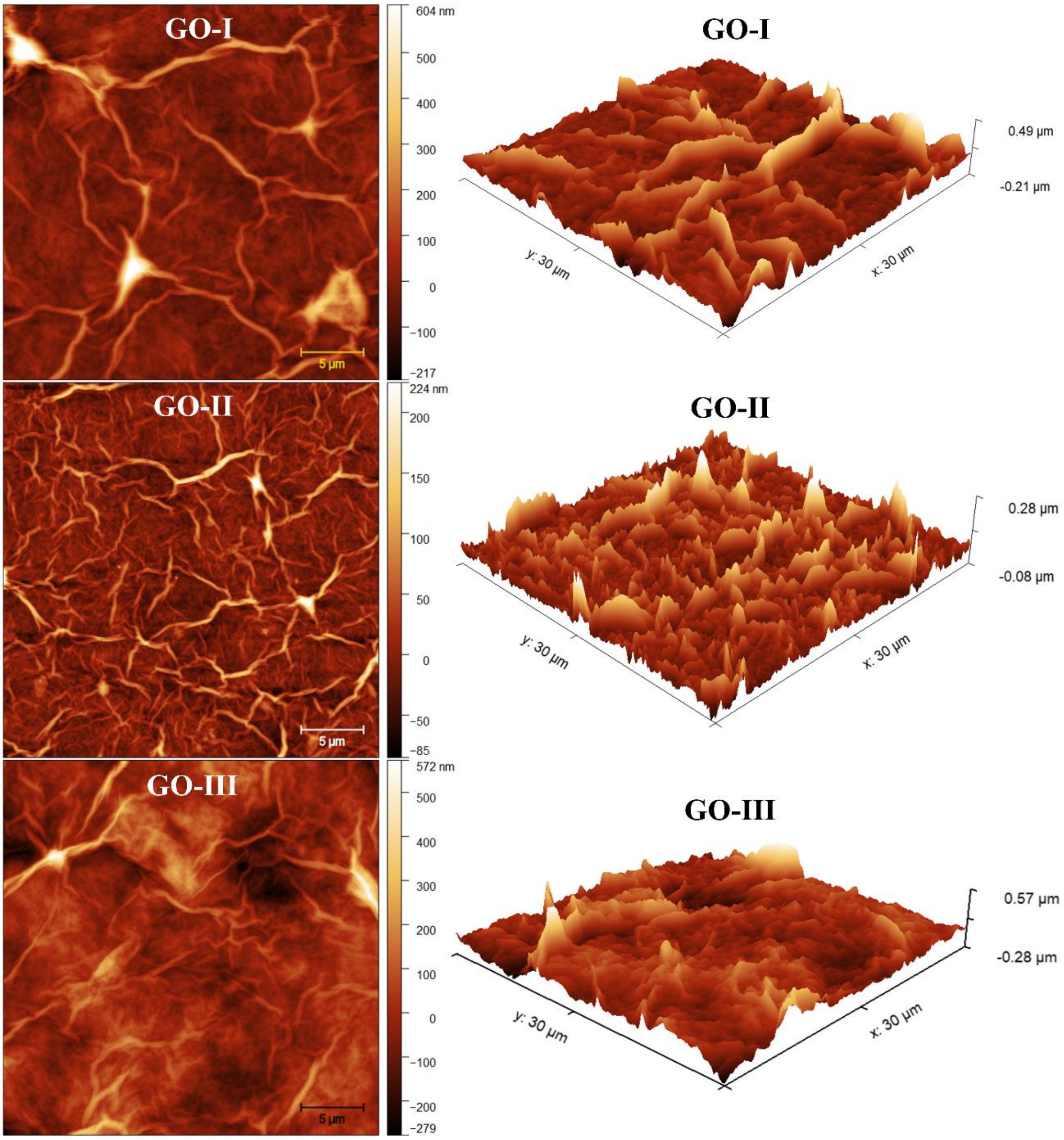

Figure S2: AFM topographic and 3D surface profile images of GO-I, GO-II, and GO-III films, acquired over a scan area of 30 µm².

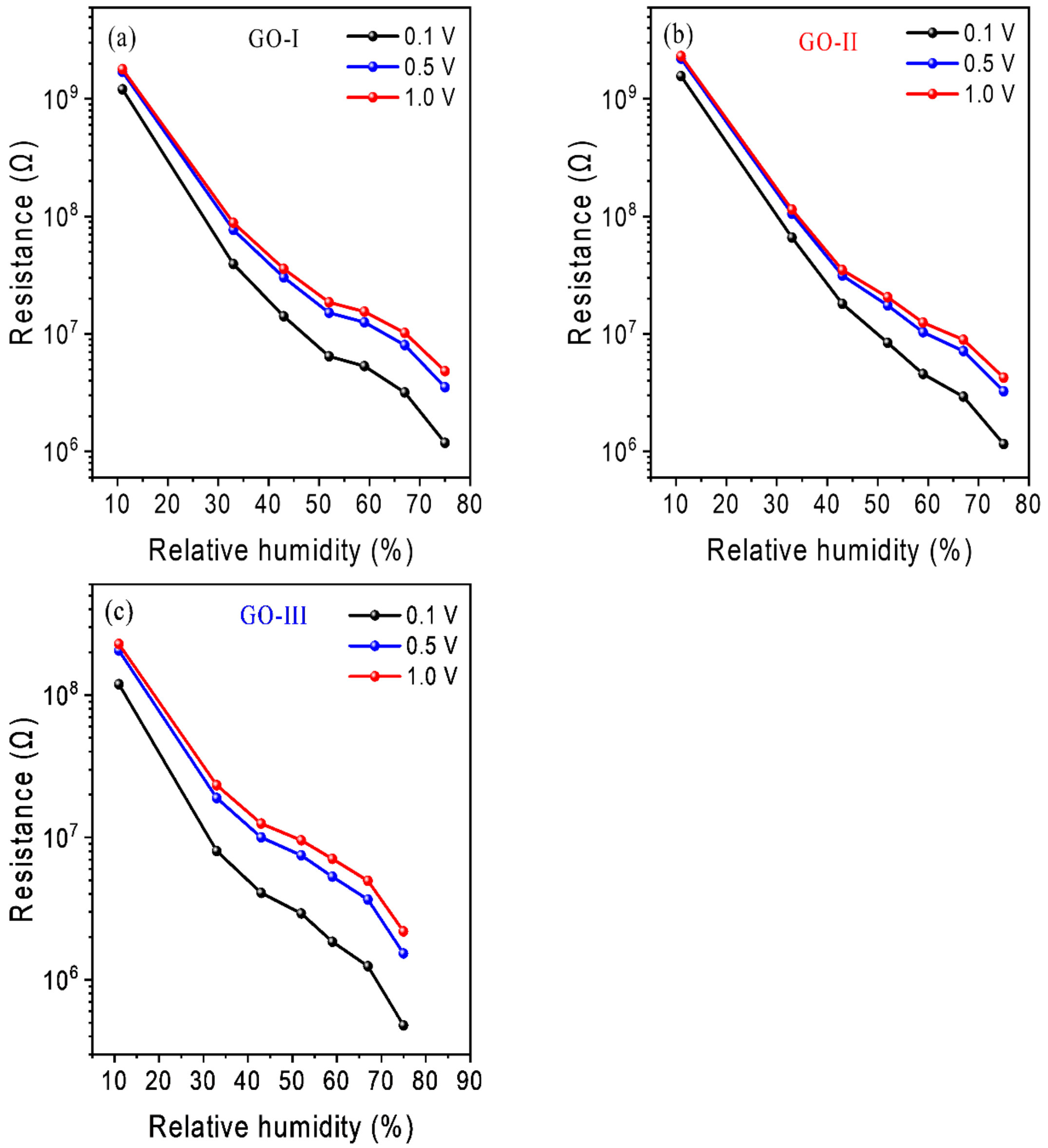

Figure S3: Logarithm of resistance as a function of RH of sensors based on (a) GO-I, (b) GO-II, and (c) GO-III, for 0.1, 0.5 and 1.0 V.

References

(1) Marcano, D. C.; Kosynkin, D. V; Berlin, J. M.; Sinitskii, A.; Sun, Z.; Slesarev, A.; Alemany, L. B.; Lu, W.; Tour, J. M. Improved Synthesis of Graphene Oxide. *ACS Nano* **2010**, *4* (8), 4806–4814. https://doi.org/10.1021/nn1006368.

(2) Almeida de Mendonça, J. P.; Lima, A. H. de; Junqueira, G. M. A.; Quirino, W. G.; Legnani, C.; Maciel, I. O.; Sato, F. Structural and Vibrational Study of Graphene Oxide via Coronene Based Models: Theoretical and Experimental Results. *Mater Res Express* **2016**, *3* (5), 055020. https://doi.org/10.1088/2053-1591/3/5/055020.

(3) Gao, W.; Alemany, L. B.; Ci, L.; Ajayan, P. M. New Insights into the Structure and Reduction of Graphite Oxide. *Nat Chem* **2009**, *1* (5), 403–408. https://doi.org/10.1038/nchem.281.

(4) Rathi, K.; Pal, K. Impact of Doping on GO: Fast Response-Recovery Humidity Sensor. *ACS Omega* **2017**, *2* (3), 842–851. https://doi.org/10.1021/acsomega.6b00399.

(5) Haidry, A. A.; Wang, Z.; Fatima, Q.; Zavabeti, A.; Xie, L.; Zhu, H.; Li, Z. Thermally Reduced Graphene Oxide Showing N- to p-Type Electrical Response Inversion with Water Adsorption. *Appl Surf Sci* **2020**, *531* (July), 147285. https://doi.org/10.1016/j.apsusc.2020.147285.

(6) Fatima, Q.; Haidry, A. A.; Yao, Z.; He, Y.; Li, Z.; Sun, L.; Xie, L. The Critical Role of Hydroxyl Groups in Water Vapor Sensing of Graphene Oxide. *Nanoscale Adv* **2019**, *1* (4), 1319–1330. https://doi.org/10.1039/c8na00135a.

(7) Li, N.; Chen, X.; Chen, X.; Ding, X.; Zhao, X. Ultrahigh Humidity Sensitivity of Graphene Oxide Combined with Ag Nanoparticles. *RSC Adv* **2017**, *7* (73), 45988–45996. https://doi.org/10.1039/c7ra06959f.

(8) Zhang, D.; Zong, X.; Wu, Z. Fabrication of Tin Disulfide/Graphene Oxide Nanoflower on Flexible Substrate for Ultrasensitive Humidity Sensing with Ultralow Hysteresis and Good Reversibility. *Sens Actuators B Chem* **2019**, *287* (November 2018), 398–407. https://doi.org/10.1016/j.snb.2019.01.123.

(9) Zhang, C.; Dabbs, D. M.; Liu, L. M.; Aksay, I. A.; Car, R.; Selloni, A. Combined Effects of Functional Groups, Lattice Defects, and Edges in the Infrared Spectra of Graphene Oxide. *Journal of Physical Chemistry C* **2015**, *119* (32), 18167–18176. https://doi.org/10.1021/acs.jpcc.5b02727.

(10) Brusko, V.; Khannanov, A.; Rakhmatullin, A.; Dimiev, A. M. Unraveling the Infrared Spectrum of Graphene Oxide. *Carbon N Y* **2024**, *229* (July), 119507. https://doi.org/10.1016/j.carbon.2024.119507.

(11) Guo, H.; Wang, X.; Qian, Q.; Wang, F.; Xia, X. A Green Approach to the Synthesis of Graphene Nanosheets. *ACS Nano* **2009**, *3* (9), 2653–2659. https://doi.org/10.1021/nn900227d.

(12) Chen, J.; Zhang, Y.; Zhang, M.; Yao, B.; Li, Y.; Huang, L.; Li, C.; Shi, G. Water-Enhanced Oxidation of Graphite to Graphene Oxide with Controlled Species of Oxygenated Groups. *Chem Sci* **2016**. https://doi.org/10.1039/c5sc03828f.

(13) Galande, C.; Mohite, A. D.; Naumov, A. V.; Gao, W.; Ci, L.; Ajayan, A.; Gao, H.;

Srivastava, A.; Bruce Weisman, R.; Ajayan, P. M. Quasi-Molecular Fluorescence from Graphene Oxide. *Sci Rep* **2011**, *1*, 1–5. https://doi.org/10.1038/srep00085.

(14) Wang, J.; Salihi, E. C.; Šiller, L. Green Reduction of Graphene Oxide Using Alanine. *Materials Science and Engineering C* **2017**, *72*, 1–6. https://doi.org/10.1016/j.msec.2016.11.017.

(15) Acik, M.; Lee, G.; Mattevi, C.; Pirkle, A.; Wallace, R. M.; Chhowalla, M.; Cho, K.; Chabal, Y. The Role of Oxygen during Thermal Reduction of Graphene Oxide Studied by Infrared Absorption Spectroscopy. *Journal of Physical Chemistry C* **2011**, *115* (40), 19761–19781. https://doi.org/10.1021/jp2052618.

(16) Pimenta, M. A.; Dresselhaus, G.; Dresselhaus, M. S.; Cançado, L. G.; Jorio, A.; Saito, R. Studying Disorder in Graphite-Based Systems by Raman Spectroscopy. *Physical Chemistry Chemical Physics* **2007**. https://doi.org/10.1039/b613962k.

(17) Ferrari, A. C.; Basko, D. M. Raman Spectroscopy as a Versatile Tool for Studying the Properties of Graphene. *Nat Nanotechnol* **2013**, *8* (4), 235–246. https://doi.org/10.1038/nnano.2013.46.

(18) Claramunt, S.; Varea, A.; López-Díaz, D.; Velázquez, M. M.; Cornet, A.; Cirera, A. The Importance of Interbands on the Interpretation of the Raman Spectrum of Graphene Oxide. *Journal of Physical Chemistry C* **2015**, *119* (18), 10123–10129. https://doi.org/10.1021/acs.jpcc.5b01590.

(19) Cançado, L. G.; Jorio, A.; Ferreira, E. H. M.; Stavale, F.; Achete, C. A.; Capaz, R. B.; Moutinho, M. V. O.; Lombardo, A.; Kulmala, T. S.; Ferrari, A. C. Quantifying Defects in Graphene via Raman Spectroscopy at Different Excitation Energies. *Nano Lett* **2011**. https://doi.org/10.1021/nl201432g.

(20) Ferrari, A.; Robertson, J. Interpretation of Raman Spectra of Disordered and Amorphous Carbon. *Phys Rev B Condens Matter Mater Phys* **2000**. https://doi.org/10.1103/PhysRevB.61.14095.
